# Identification of a stable major-effect QTL (Parth 2.1) controlling parthenocarpy in cucumber and associated candidate gene analysis via whole genome re-sequencing

**DOI:** 10.1186/s12870-016-0873-6

**Published:** 2016-08-23

**Authors:** Zhe Wu, Ting Zhang, Lei Li, Jian Xu, Xiaodong Qin, Tinglin Zhang, Li Cui, Qunfeng Lou, Ji Li, Jinfeng Chen

**Affiliations:** 1 State Key Laboratory of Crop Genetics and Germplasm Enhancement, Nanjing Agricultural University, Nanjing, 210095 China; 2 College of Horticulture, Shanxi Agricultural University, Shanxi, 030801 China

**Keywords:** Parthenocarpy, Cucumber, QTL, Re-sequencing, Candidate genes

## Abstract

**Background:**

Parthenocarpy is an important trait for yield and quality in many plants. But due to its complex interactions with genetic and physiological factors, it has not been adequately understood and applied to breeding and production. Finding novel and effective quantitative trait loci (QTLs) is a critical step towards understanding its genetic mechanism. Cucumber (*Cucumis sativus* L.) is a typical parthenocarpic plant but the QTLs controlling parthenocarpy in cucumber were not mapped on chromosomes, and the linked markers were neither user-friendly nor confirmed by previous studies. Hence, we conducted a two-season QTL study of parthenocarpy based on the cucumber genome with 145 F_2:3_ families derived from a cross between EC1 (a parthenocarpic inbred line) and 8419 s-1 (a non-parthenocarpic inbred line) in order to map novel QTLs. Whole genome re-sequencing was also performed both to develop effective linked markers and to predict candidate genes.

**Results:**

A genetic linkage map, employing 133 Simple Sequence Repeats (SSR) markers and nine Insertion/Deletion (InDel) markers spanning 808.1 cM on seven chromosomes, was constructed from an F_2_ population. Seven novel QTLs were identified on chromosomes 1, 2, 3, 5 and 7. Parthenocarpy 2.1 (Parth2.1), a QTL on chromosome 2, was a major-effect QTL with a logarithm of odds (LOD) score of 9.0 and phenotypic variance explained (PVE) of 17.0 % in the spring season and with a LOD score of 6.2 and PVE of 10.2 % in the fall season. We confirmed this QTL using a residual heterozygous line97-5 (RHL97-5). Effectiveness of linked markers of the Parth2.1 was validated in F_3:4_ population and in 21 inbred lines. Within this region, there were 57 genes with nonsynonymous SNPs/InDels in the coding sequence. Based on further combined analysis with transcriptome data between two parents, *CsARF*19, *CsWD40*, *CsEIN1*, *CsPPR*, *CsHEXO3, CsMDL, CsDJC77* and *CsSMAX1* were predicted as potential candidate genes controlling parthenocarpy.

**Conclusions:**

A major-effect QTL Parth2.1 and six minor-effect QTLs mainly contribute to the genetic architecture of parthenocarpy in cucumber. SSR16226 and Indel-T-39 can be used in marker-assisted selection (MAS) of cucumber breeding. Whole genome re-sequencing enhances the efficiency of polymorphic marker development and prediction of candidate genes.

**Electronic supplementary material:**

The online version of this article (doi:10.1186/s12870-016-0873-6) contains supplementary material, which is available to authorized users.

## Background

Parthenocarpy is defined as fruit set in the absence of fertilization or other stimulation [1]. It has the potential to increase yield, especially under unfavorable conditions, e.g. in protected cultivation. Moreover, parthenocarpic fruits tend to be firmer and fleshier than seeded ones [2]. Therefore, development of parthenocarpy cultivars is one of the most important targets in plant breeding.

Parthenocarpy can be influenced by environmental, physiological, and genetic factors. Environmental conditions such as low temperatures and short day lengths promote parthenocarpy. Parthenocarpy has been shown to be dependent certain hormones. For instance, endogenous IAA concentrations in parthenocarpic ovaries or on fruits have been found to be higher than in pollinated organs in cucumbers [3–5]. There is also evidence that exogenous plant growth-regulating chemical, including auxin and auxin transport inhibitors, gibberellin, cytokinin, and brassinosteroids can induce parthenocarpy [6–10]. Parthenocapy fruit set can be induced with the application of compatible foreign pollen to stigma [11–13] because pollen contains auxins, gibberellins, and brassinosteroids [13, 14]. Moreover, introducing the *DefH9*-*iaaM* auxin-synthesizing gene into cucumber [15], eggplant and tobacco [16] can stimulate parthenocarpy. Overexpression of *SLTIR1* (an auxin receptor) [17], down-regulated expression of *SLARF7* (Auxin Response Factor 7) [18] and *SLIAA9* (a subfamily of Aux/IAA gene) transgenic tomatoes [19] also give rise to parthenocarpy. Genetic analyses have led to the successful identification of some genes associated with parthenocarpy in tomato and eggplant. In tomatoes, eight parthenocarpic genes—pat, pat-2, pat-3/pat-4, pat4.1/pat5.1, and pat4.2/pat9.1 were identified. Among them, pat, pat4.1, pat4.2, pat5.1 and pat9.1 were mapped on genetic linkage maps [20, 21]. In eggplant, QTL analyses revealed two QTLs on chromosome 3 and on chromosome 8, which were denoted as *Controlling parthenocarpy3.1 (Cop3.1)* and *Cop8.1*, respectively [22].

Parthenocarpy is widespread in cucumber germplasm resources, and so cucumber is a promising model plant for the study of parthenocarpy. Genetic studies of parthenocarpy in cucumber started in 1930. Hawthorn [23], Juldasheva [24], and Meshcherov [25] found that parthenocarpy in cucumber is controlled by one recessive gene, whereas Kvasnikov [26], using a European processing type, proposed that many incompletely recessive genes are responsible for controlling parthenocarpy. Kim and Pike [3, 27] report that a single incompletely dominant gene controlled parthenocarpy. Ponti and Peterson [28], conducting an incomplete diallel cross between different pickling cucumber lines, came to the conclusion that three independent, isomeric major genes, control parthenocarpy in conjunction with additive genes. While most recent studies suggest that inheritance of parthenocarpy in cucumber is consistent with characteristics of quantitative traits [29–32], and Sun [33] identified ten QTLs associated with parthenocarpy distributed across four genomic regions as well as eight linked AFLP markers in cucumber. However, the location of these QTLs on the chromosomes is still unknown, and the related linked markers have neither been confirmed nor been shown to be breeder friendly. Hence, QTL mapping of parthenocarpy based on cucumber genome is needed as a means of finding novel QTLs and developing effective linked markers. Traditional QTL analysis approaches are laborious and time-consuming due to less polymorphic markers for map construction and difficulties of candidate gene prediction. Whole genome sequencing methods can overcome these limitations. For example, researchers have used whole genome re-sequencing to genotype [34] or to QTL-seq [35], thereby speeding up the process of QTL mapping.

In this study, we performed a two-season QTL study for parthenocarpy in cucumber in F_2:3_ families from an EC1 × 8419 s-1 cross. The major-effect QTL was confirmed with RHL97-5 (a residual heterozygous line97-5). The effectiveness of linked markers to this QTL was validated in F_3:4_ plants and in 21 inbred lines. Whole genome re-sequencing allowed us to develop polymonrphic markers and predict candidate genes. The ascertainment of the major-effect QTL of parthenocapy will provide a good foundation for its fine mapping with large segregating population and the linked markers to this QTL will be useful for molecular breeding of parthenocarpy in cucumber.

## Results

### Evaluation of parthenocarpy ability

**Fig. 1:**
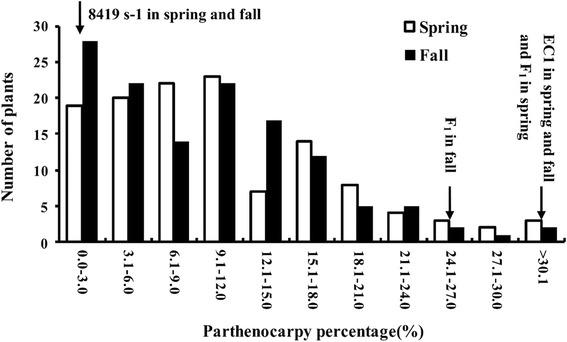
Frequency distribution of PP means of F_2:3_ families in spring and fall 2013

The phenotypic means, standard deviation and range of parthenocarpy from two seasons are presented in Table 1 which is based on simple averages of observations. All phenotype data in our study were arcsin transformed. Parthenocarpy percentage (PP) means of EC1 in spring and fall in 2013 were 51.41 and 45.40 respectively (Table 1). 8419 s-1, by comparison, aborted easily and showed extremely low PP (4.44). F_1_ derived from these two parents exhibited medium PP (37.11 and 31.37). Results from ANOVA and variance component analysis for parthenocarpy from the F_2:3_ population are presented in Additional file 1: Tables S1 and Table 2 respectively. F_2:3_ family in two seasons both revealed significant difference between F_2:3_ families (*F* value = 6.85, *P* < 0.0001), seasons (*F* value = 7.03, *P* < 0.05), and family × season interactions (*F* value = 1.62, *P* < 0.0001). The broad sense heritability estimate (*h*^*2*^) for parthenocarpy was 78.3 %. A significant positive correlation (*r* = 0.59, *P* < 0.001) (Additional file 2) was also found between PP of F_2:3_ family in different environments. The frequency distribution of PP in F_2:3_ in both seasons was a continuous distribution skewed towards non-parthenocarpy (Fig. 1). These results indicate that parthenocarpy is a quantitative trait significantly affected by environment and PP means of families in different seasons could be used for subsequent QTL analyses.

**Table 1:** Phenotypic means and range of parthenocarpy in two parental lines (EC1, 8419 s-1), their F_1_ and 123 F_2:3_ families in spring and fall in 2013

Season	EC1	8419 s-1	F_1_	F_2_:_3_ Family	F_2_:_3_ Family
	Mean ± SD	Mean ± SD	Mean ± SD	Mean ± SD	Range
Spring	51.41 ± 17.26	4.44 ± 8.13	37.11 ± 11.97	18.91 ± 15.79	0–35.24
Fall	45.40 ± 15.23	4.44 ± 8.13	31.37 ± 9.80	18.05 ± 15.56	0–34.02

Phenotypic data were evaluated by parthenocarpy percentage (PP) that was arcsin transformed

**Table 2:** Variance components and broad heritability estimates based on F_2:3_ data

Variance components	PP
σ^2^_F_	39.30
σ^2^_FS_	9.55
σ^2^_E_	123.36
Heritability (h^2^_B_)	0.783

σ^2^_F_ is the family variance, σ^2^_FS_ is the family × season interaction (F × S) variance, and σ^2^_E_ is the residual variance

### Genetic map construction and QTL mapping

After screening 1335 SSR markers and 173 InDel markers between two parental lines, we identified 232 polymorphic pairs (15.4 %). Some markers that didn’t show good amplification products or segregate in F_2_ plants were deleted. Among them, 133 SSR markers and 9 Indel markers were successfully mapped (Additional file 3). Most of markers fit the expected 1:2:1 segregation ratio, with the exception of 28 markers (19.7 %) (those with asterisk in Additional file 1: Table S2), which exhibited distorted segregation in *χ*^2^ tests (*P* < 0.05). The map covered a total of 808.1 cM and contained 7 chromosomes. The number of markers on each chromosome was between 14 and 26, and the average marker interval of this map was 5.7 cM (Additional file 1: Table S3). Most of marker orders were well consistent with their physical position in 9930 genome (Additional file 1: Table S2), so we used this linkage map to detect QTLs for parthenocarpy in cucumber.

**Fig. 2:**
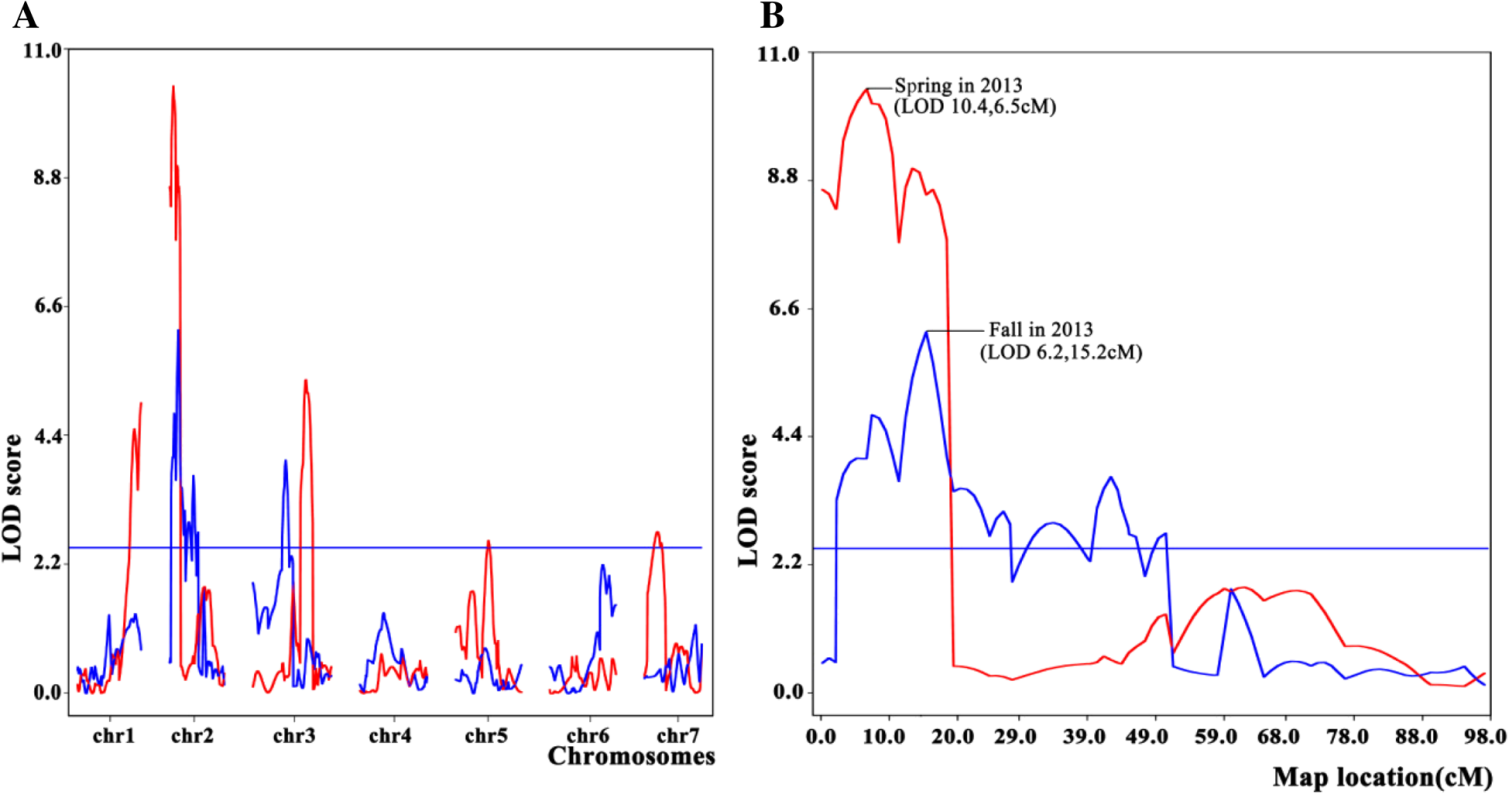
QTL mapping of parthenocarpy based on phenotypic data in spring and fall 2013. **a**. All QTLs detected in seven chromosomes. **b**. LOD curves of the QTL on chromosome 2

Seven QTLs for parthenocarpy were detected on chromosomes 1, 2, 3, 5, and 7 on the basis of the PP means of F_2:3_ families in spring and fall 2013 (Fig. 2a; Additional file 3, Table 3). The additive effects of QTLs on chromosomes 1, 2, and 3 were positive, which indicated the alleles that increase PP come from EC1, whereas QTLs on chromosome 5 and 7 had negative additive effects and the alleles that increase PP come from 8419 s-1. In spring, five QTLs were detected including Parth1 at 101.0 cM (LOD 4.5, *R*^*2*^ = 7.8 %) of chromosome 1, Parth2.1 at 6.5 cM (LOD 10.4, *R*^*2*^ = 17.0 %) of chromosome 2, Parth3.1 (LOD 5.3, *R*^*2*^ = 6.4 %) at 93.8 cM of chromosome 3, Parth5 (LOD 2.6, *R*^*2*^ = 4.1 %) at 58.0 cM of chromosome 5, Parth7 (LOD 2.8, *R*^*2*^ = 8.9 %) at 23.4 cM of chromosome 7 (Table 3). We detected three QTLs in fall: Parth2.1 (LOD 6.2 *R*^*2*^ = 10.2 %), Parth2.2 at 50.3 cM (LOD3.6, *R*^*2*^ = 7.2 %) of chromosome 2 and Parth3.1 at 57.5 cM (LOD 4.0, *R*^*2*^ = 5.2 %) of chromosome 3. Parth2.1 flanked by SSR00684 and SSR22083 was considered as a major-effect QTL since it was the only QTL detected in two seasons and could explain more than 10 % of the phenotypic variance (Fig. 2b; Additional file 3)

**Table 3:** QTLs for parthenocarpy of cucumber detected in EC1//8419 s-1 F_2:3_ families in spring and fall 2013

Season	QTL	Chromosome	Peak(cM)	LOD	*R* ^*2*^	Additive effect	Dominance effect	Marker interval
Spring	Parth1	1	101.0	4.5	7.8	3.5	0.3	UW085142-SSR00262
	Parth2.1	2	6.5	10.4	17.0	5.3	0.7	SSR00684-SSR22083
	Parth3.2	3	93.8	5.3	6.4	3.9	1.4	SSR03621-UW085093
	Parth5	5	58.0	2.6	4.1	−2.7	−0.3	SSR03341-SSR19178
	Parth7	7	23.4	2.8	8.9	−2.9	2.2	SSR30647-SSR04689
Fall	Parth2.1	2	15.2	6.2	10.2	4.1	2.5	SSR00684-SSR22083
	Parth2.2	2	50.3	3.6	7.2	4.2	0.1	Indel-68-UW085299
	Parth3.1	3	57.5	4.0	5.2	3.5	1.3	SSR17751-UW084149

### Confirmation of the major-effect QTL, Parth2.1

**Fig. 3:**
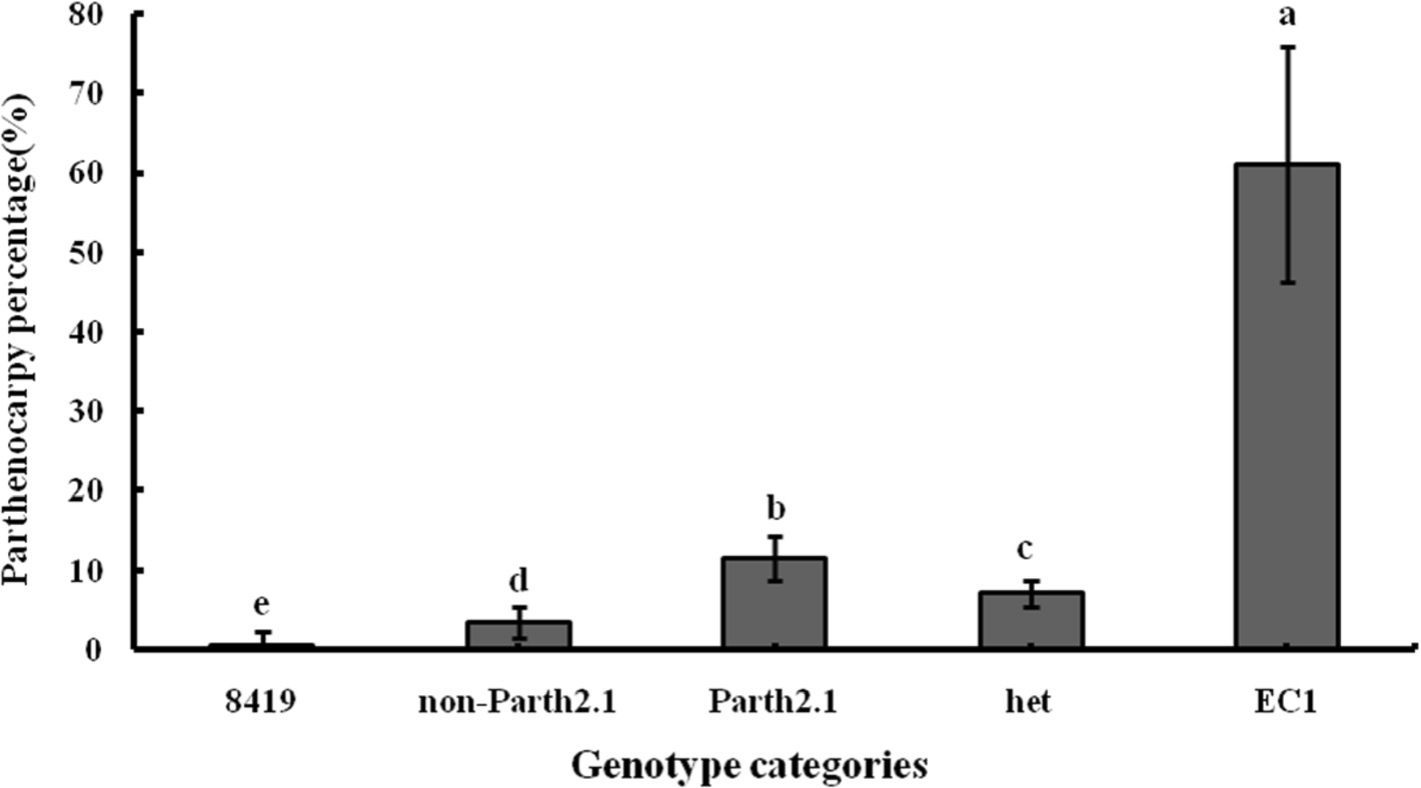
Confirmation of the Parth2.1 based on genotype of 161 plants in Parth2.1 region. Each bar is the mean parthenocary percentage of each category. Error bars represent the t value * standard errors of each category with t value from a student-t table. The distinct letters show significance at *P* < 0.05 based on ANOVA

We confirmed the presence of Parth2.1 with 161 plants of RHL97-5 segregating for Parth2.1 (Fig. 3). Plants carrying homozygous alleles of EC1 in Parth2.1 region have significantly higher PP (11.57 ± 1.36) compared to those with homozygous 8419 s-1 alleles (3.50 ± 0.96) at *P* < 0.05. Similarly, plants harboring the heterozygous alleles of the QTL (7.16 ± 0.85) were statistically significantly higher than those containing homozygous 8419 s-1 alleles but significantly lower than those with homozygous EC1 alleles at *P* < 0.05. These results confirmed the QTL effect, with 8.07 % higher PP for plants containing the homozygous EC1 alleles over plants with homozygous 8419 s-1 alleles at Parth2.1. Moreover, PP of the donor parent EC1 (61.11 ± 6.57) was significantly higher than plants having homozygous EC1 alleles in the Parth2.1 QTL region (*P* < 0.05), implying that the other QTLs also contributed to parthenocarpy in addition to Parth2.1.

**Fig. 4:**
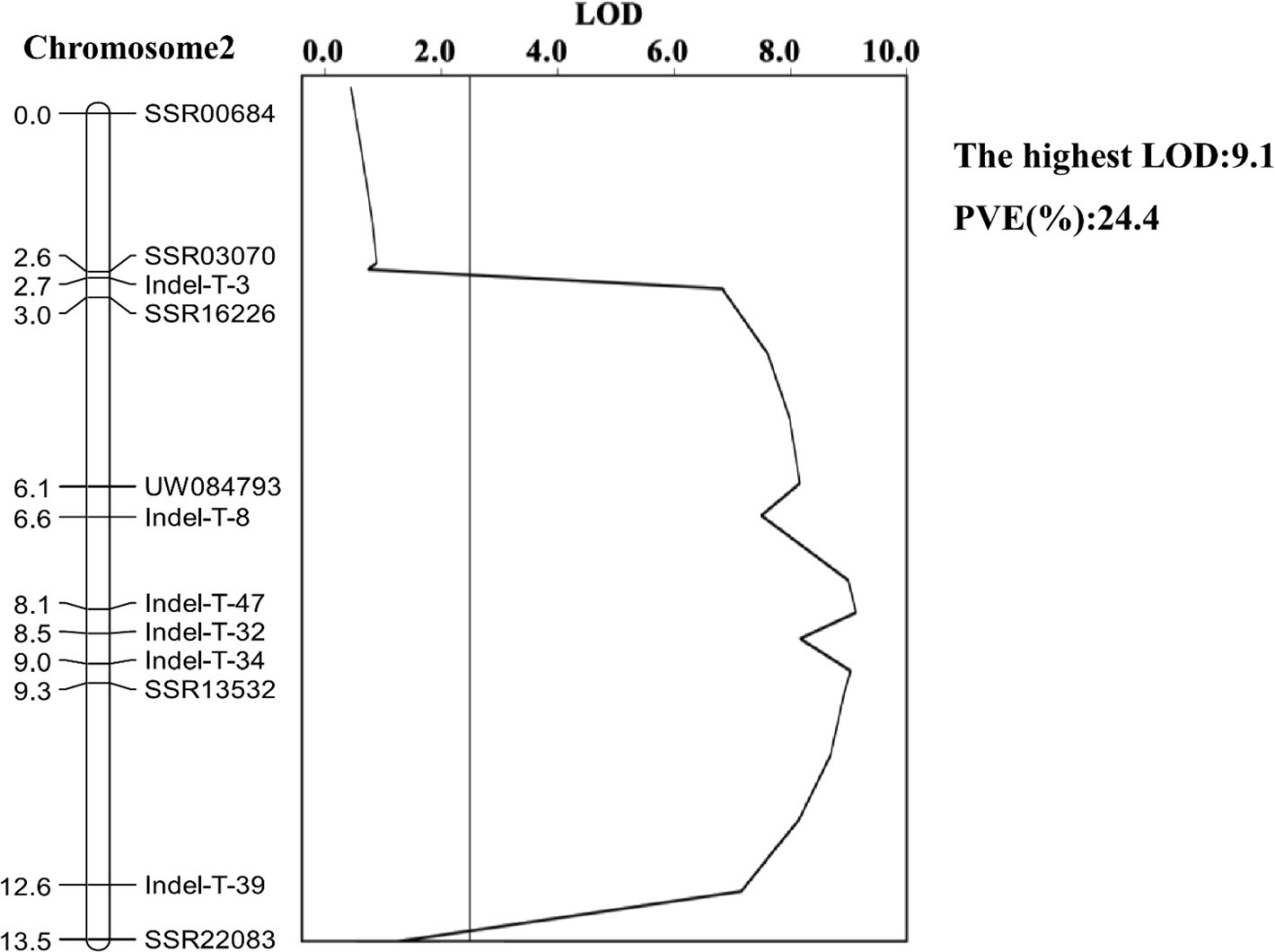
High-resolution genetic map in Parth2.1 region and QTL analysis results based on 161 plants

A linkage map of Parth2.1 with a genetic distance of 13.5 cM was constructed based on genotyping of 161 plants of RHL97-5 with 6 SSR markers and 6 newly developed InDel markers (Fig. 4). This linkage map was shorter than the map constructed by F_2_ population (17.1 cM) and the mean distance between two neighboring markers was 1.09 cM. Linkage mapping analysis showed a major-effect QTL of parthenocarpy with a PVE of 24.4 %. The highest LOD score of 9.1 located between SSR16226 and Indel-T-39 according to a 2-LOD drop for a confidence interval of the QTL (Fig. 4), verifying that the QTL was very likely located in this region.

### Validation of the effectiveness of the markers linked to Parth2.1

Indel-T-32, Indel-T-34 and two flanking markers, SSR16226 and Indel-T-39 of Parth2.1, were used to genotype 99 F_3:4_ plants. We classified these plants into three groups according to their genotypes. *χ*^2^ test results of Indel-T-32, Indel-T-34, SSR16226 and Indel-T-39 were *χ*^2^ = 20.13 > χ^2^_0.01,8_(20.09), *χ*^2^ = 19.20 > χ^2^_0.05,8_(15.51), *χ*^2^ = 25.73 > χ^2^_0.01,8_(20.09) and *χ*^2^ = 17.59 > χ^2^_0.05,8_(15.51) respectively indicating that these markers were significantly related to parthenocarpy. The PP means of plants with homozygous EC1 alleles at loci Indel-T-32, Indel-T-34, SSR16226 and Indel-T-39 were 26.84 ± 11.86, 26.89 ± 11.76, 26.80 ± 11.78 and 27.89 ± 11.41 respectively which were significantly higher than those plants with homozygous 8419 s-1 alleles (19.54 ± 11.72, 19.04 ± 11.80, 13.72 ± 9.97 and 19.54 ± 11.72) at *P* < 0.01. The PP means of plants with heterozygous genotype at loci Indel-T-32, Indel-T-34 and Indel-T-39 were significantly lower than those with homozygous EC1 alleles at *P* < 0.05 but not significantly different with those with homozygous 8419 s-1 alleles whereas at locus SSR16226 showed the opposite way (Table 4).

**Table 4:** PP means for 99 F_3:4_ plants with different genotypes at SSR16226, Indel-T-32, Indel-T-34 and Indel-T-39 loci

Genotype	SSR16226	Indel-T-32	Indel-T-34	Indel-T-39
EC1 type	26.80 ± 11.78aA(55)	26.84 ± 11.86aA(54)	26.89 ± 11.76aA(55)	27.89 ± 11.41aA(50)
8419 s-1 type	13.15 ± 10.13bB(33)	19.54 ± 11.72bB(36)	19.04 ± 11.80bB(34)	16.58 ± 11.99bB(42)
Heterozygous type	25.40 ± 16.06aA(11)	15.63 ± 16.08bAB(9)	15.24 ± 15.24bAB(10)	13.82 ± 15.32bB(7)

The lower case letter indicates significance at *P* < 0.05, and the capital letter indicates significance at *P* < 0.01. Numbers in brackets are numbers of plants based on different genotypes

**Fig. 5:**
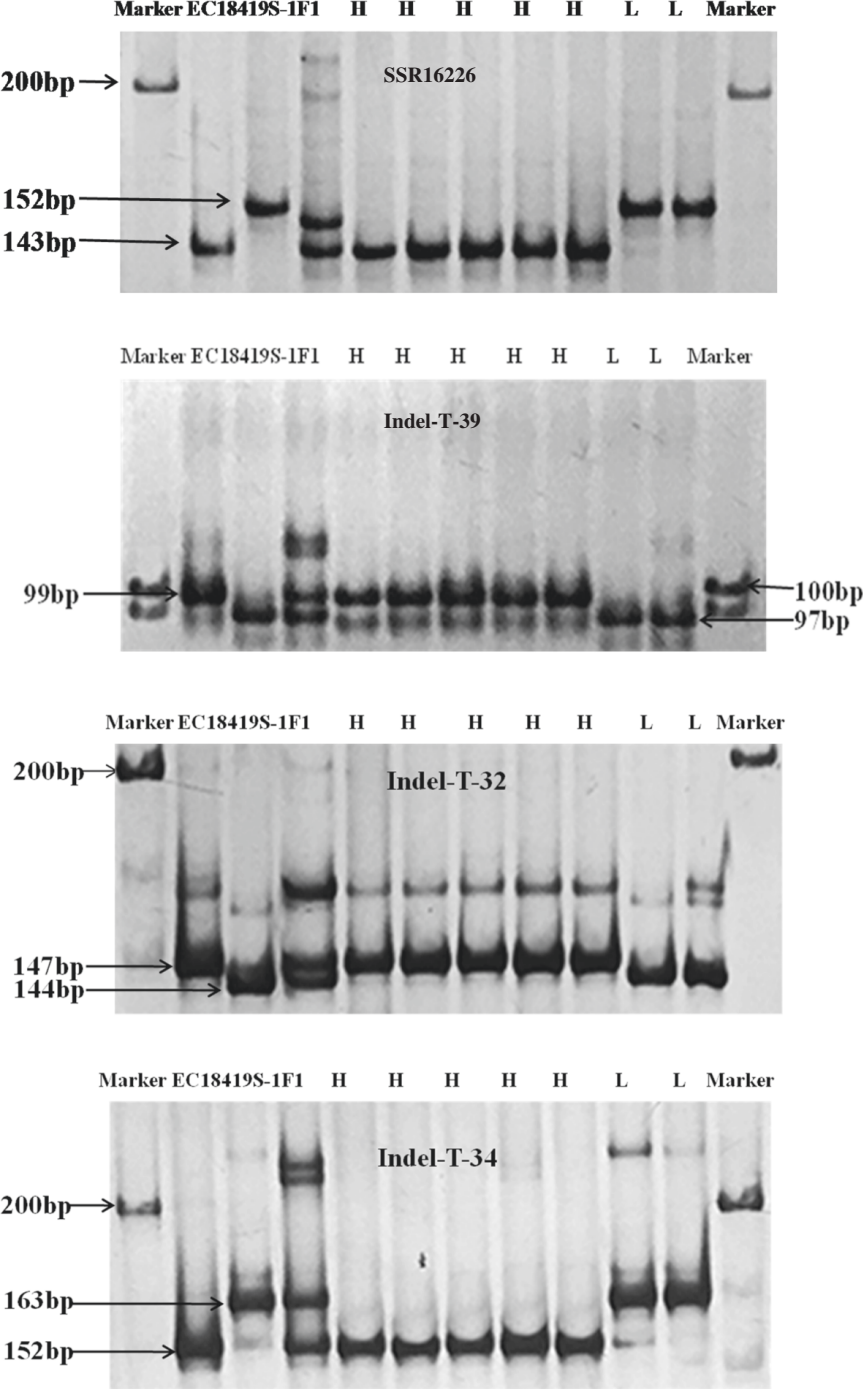
Amplification products produced by markers SSR16226, Indel-T-32 Indel-T-34 and Indel-T-39 in cucumber inbred lines. H represents high PP inbred lines that were 14405, 14438, 14422, 14496, 14427 respectively, and N represents non-parthenocarpy inbred lines that were 14418 and 14435 respectively

We also collected phenotype data of 11 gynoecious and 10 monoecious cucumber inbred lines (Additional file 1: Table S4) and genotyped them with SSR16226, Indel-T-32, Indel-T-34 and Indel-T-39. The amplification products of these markers of five gynoecious inbred lines (14405, 14438, 14422, 14496, 14427) with high PP (higher than F_1_) and two gynoecious non-parthenocapic inbred lines (14418 and 14435) after electrophoresis are shown in Fig. 5. Five high PP inbred lines all showed the same band with EC1, whereas two non-pathenocarpic inbred lines showed the same band with 8419 s-1. In contrast to gynoecious inbred lines, monoecious inbred lines exhibited low PP and these markers did not show any relationship with parthenocarpy of these lines (data not shown).

### Analysis of candidate genes based on re-sequencing and RNA-seq of two parents

We carried out whole genome re-sequencing of the two parents to obtain polymorphism data set (see “methods”). The polymorphic nucleotide sequences between EC1 and 8419 s-1, including InDels, were obtained by comparing the whole genome sequences of EC1 and 8419 s-1 with the reference ‘9930’ sequence. There were 83,119 SNPs and 14,772 InDels in EC1, 52,278 SNPs and 9462 InDels in 8419 s-1 on chromosome 2 (Additional file 1: Table S5).

Referring to the cucumber genome database (http://cucumber.genomics.org.cn/page/cucumber/index.jsp), 241 genes located within the Parth2.1 region. By comparing the whole genome sequences of EC1 and 8419 s-1 with the reference 9930 sequence, we found 57 candidate genes containing the polymorphic SNP/Indels in the coding sequence regions that led to missense or frameshift mutations (Additional file 1: Table S6). We further investigated the orthologs of these candidate genes in *Arabidopsis thaliana* using TAIR (http://www.arabidopsis.org/) databases. Most of them have been functionally characterized (Additional file 1: Table S6). Three of 57 genes, Csa2M068680 (*CsARF*19), Csa2M070230 (*CsWD40*) and Csa2M070880 (*CsEIN1*) were identified as phytohormone related genes. Csa2M068680 (*CsARF19*) encodes AUX/IAA like protein, which functions in various biological processes, e.g. lateral root development, fruit development [19, 36, 37]. The tomato Aux/IAA transcription factor IAA9 is involved in fruit development and leaf morphogenesis [19]. The *Solanum lycopersicum* auxin response factor 7 (*SlARF7*) regulates auxin signaling during tomato fruit set and development [18]. Csa2M070230 (*CsWD40*) encodes WD-40 repeat family protein, which functions in cytokinin responses [38, 39]. Csa2M070880 (*CsEIN1*) encodes prokaryote sensory transduction proteins, which functions in ethylene binding and has ethylene receptor activity [40–42].

**Fig. 6:**
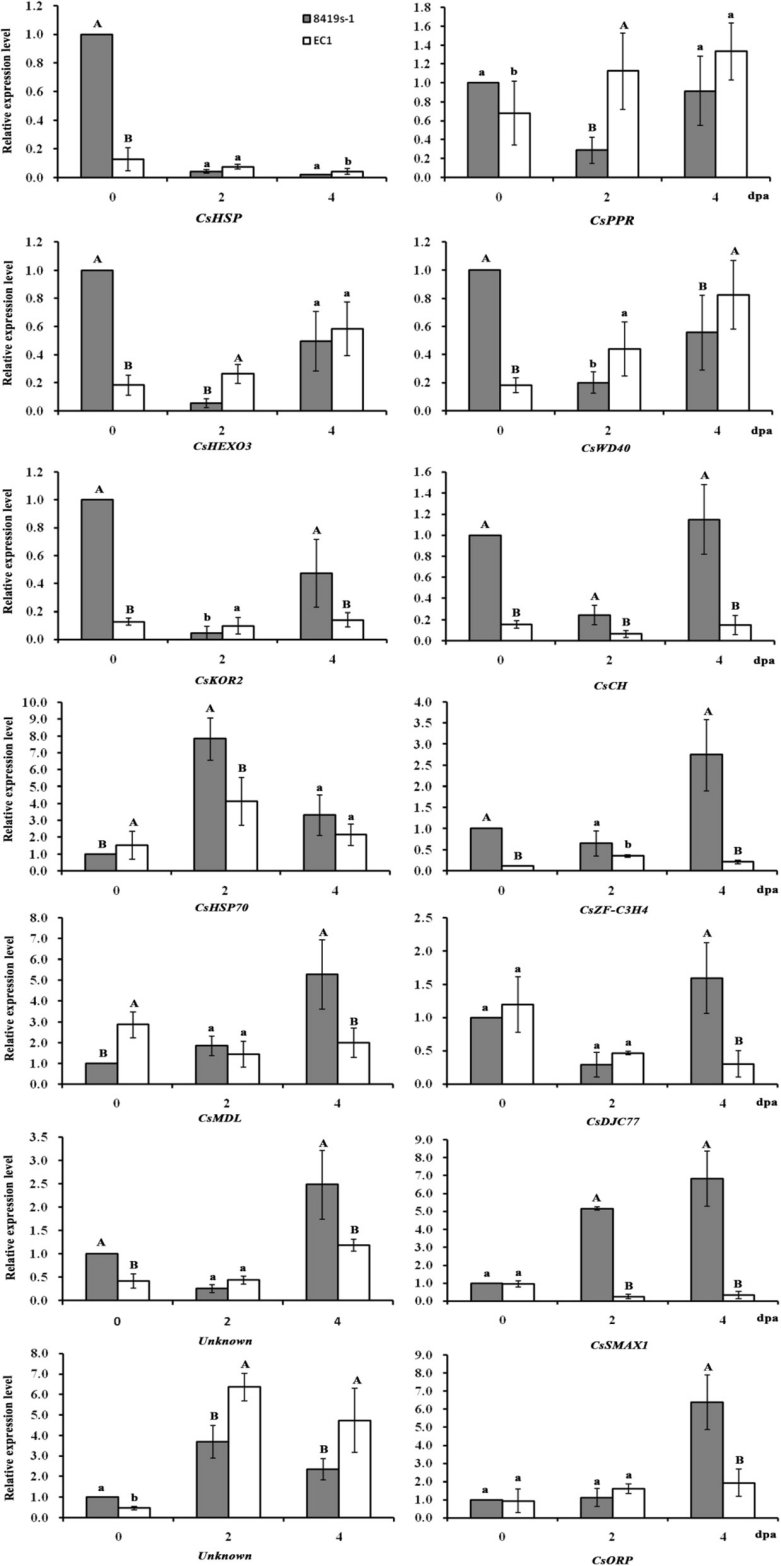
Expression level of 14 genes by quantitative real-time PCR. a, b and A, B indicate the least significant difference at 0.05 and 0.01 between EC1 and 8419 s-1 at corresponding day post anthesis (dpa) respectively. Values are the mean ± t * SE, with t value from a student-t table

Furthermore, we used the transcriptome data within the Parth2.1 [43] and found that 14 genes were differentially expressed between parthenocapic fruit of EC1 and abortive fruit of 8419 s-1 (the false discovery rate ≤ 0.001 and the fold ≥1.5) (Additional file 1: Table S7). Interestingly, the phytohormone related genes Csa2M070230 (*CsWD40*) also expressed differentially. Moreover, qRT-PCR suggested that transcription of Csa2M070230 (*CsWD40*), Csa2M070330 (*CsPPR*) and Csa2M073000 (*CsHEXO3*) were continuously up-regulated whereas Csa2M055050 (*CsMDL*), Csa2M058620 (*CsDJC77*) and Csa2M058620 (*CsSMAX1*) were continuously down-regulated during the parthenocarpic fruit set (Fig. 6). Csa2M070330 (*CsPPR*) encodes a pentatricopeptide repeat protein involved in mitochondrial RNA editing. Csa2M073000 (*CsHEXO3*) encodes a protein with beta-hexosaminidase activity. Csa2M055050 (*CsMDL*) encodes VHS domain-containing protein or GAT domain-containing protein involved in cyanide biosynthetic process. Csa2M058620 (*CsDJC77*) encodes DNA heat shock N-terminal domain-containing protein involved in protein folding. Csa2M058640 (*CsSMAX1*) encodes heat shock related-protein involved in protein metabolic process. Compared to 8419 s-1, Csa2M070330 (*CsPPR*) and Csa2M073000 (*CsHEXO3*) showed significant expression at *P* < 0.01 at 2 dpa in EC1, Csa2M070230 (*CsWD40*) and Csa2M058640 (*CsSMAX1*) showed significant expression at *P* < 0.05 and 0.01 at 2 and 4 dpa respectively in EC1 (Fig. 6), which were in accordance with transcriptome data (Additional file 1: Table S7). Obviously, *CsHEXO3* and *CsWD40* were identified by both coding sequence (Additional file 1: Table S6) and qRT-PCR analysis (Fig. 6).

## Discussion

### Map construction

It is widely known that cucumber has a narrow genetic base [44], which results in low polymorphism among cultivars. This can be seen from the marker polymorphism between two parents (15.4 %) in this study. In particular, chromosome 2 cannot be well covered with published SSR markers. As a result, we used 173 InDel markers on chromosome 2 developed by re-sequencing to screen polymorphic markers and nine of them were assigned to the target region. Almost one fifth of the mapped markers deviated from the expected segregation ratio, with some small distorted segregation clusters on chromosomes 2 and 6. To test their effects on the linkage map, we constructed the map with or without these deviated markers. Finally, we found that marker orders and intervals were not influenced by them. Segregation distortion and marker clustering have been reported in cucumber [45–47] but the reason for these phenomena is yet unclear. It is difficult to compare the map constructed by Sun [33] with the map constructed in this study due to different parents and marker types. Although it’s not a high-resolution linkage map, it’s enough for QTL mapping with mapping population size of 100–200 [48] because QTL detection power cannot be improved with the increase of the marker dense when the mean marker interval is 5–10 cM [49].

### QTLs for parthenocarpy in cucumber

Expression of multiple genes is influenced by the environment. Therefore, it is necessary to identify stable QTLs in different environments by using segregated populations. In this study, the values of PP means of donor parent and F_1_ were much higher in spring than in fall. ANOVA showed significant family (genotype) × season interaction differences (*P* < 0.001) as well, which is consistent with the conclusions drawn by Sun [33] and Kikuchi [50] that environment significantly affects expression of parthenocarpic genes. The PP means among the F_2:3_ families in two seasons also exhibited wide genetic variations (low PP means with large standard derivation among F_2:3_ families) (Table 1) and continuous distribution within the range of 0–33.3 % (or 31.3 %) (Fig. 1). Moreover, the close correlation of PP means of F_2:3_ families between two seasons (Additional file 2) demonstrated that there was a stable association between phenotype and genotype of parthenocarpy. Thus, using these phenotype data in two seasons can detect stable and environment-dependent QTLs for parthenocarpy.

We identified five significant QTLs in spring and three in fall in this study. Five of these QTLs showed positive additive effects, which indicated that alleles increasing PP come from high parthenocarpic parent EC1. However, parent 8419 s-1 also carried the alleles increasing PP on two QTLs of Parth5.1 and Parth7.1 that could explain why 8419 s-1 produced parthenocarpic fruits in some plants although PP is pretty low. Therefore, the linked markers at Parth5.1 and Parth7.1 from 8419 s-1 should be used during MAS for parthenocarpy in cucumber. The QTL Parth2.1 on chromosome 2, which contributed over 10 % of PVE and expressed in both seasons, was a stable and major-effect QTL. The rest of QTLs were environment-specific with low PVE, indicating that a major and many minor effects mainly contribute to the genetic component of parthenocarpy in cucumber. A study has been carried out for QTL mapping of parthenocarpy in cucumber. Sun [33] detected 10 QTLs in four genomic regions by using F_2:3_ families derived from a cross between two U.S. processing type of lines, however, these QTLs were not mapped on chromosomes and thus difficult to infer their locations to the map constructed in this study. Therefore, all QTLs detected in this study were novel parthenocarpic loci. Although Parth2.1 was detected in both seasons, the multiple peaks of the LOD curves in this QTL region made it difficult to find the exact QTL (Fig. 2b). The reason might be the moderate-sized population for phenotypic collection (125–130 F_2:3_ families) and moderate marker density that provide less opportunities for recombination and subsequently limit the precision of QTL detection. To improve this situation, a high resolution map in the target region and an advanced population segregating only in this region will be beneficial.

QTL confirmation is an indispensable step to make sure a target QTL that can be further studied and to measure its effect more accurately. Using a segregated population, RHL97-5, the major-effect QTL Parth2.1 was confirmed in a homozygous background at other QTLs (Fig. 3). Parth2.1 provided a 8.07 % increase in PP in contrast to non-Parth2.1 alleles at Parth2.1, which was significant at *P* < 0.05. Likewise, PP of plants with homozygous EC1 alleles was significantly higher than those with the heterozygous genotype in the QTL region, suggesting a dominance effect, in contrast to the original QTL study which showed a larger additive effect for Parth2.1.

Based on the re-sequencing information of two parents, we developed new InDel markers to construct a high-resolution linkage map in Parth2.1 region. Linkage mapping analysis revealed a major QTL with higher PVE of 24.4 % compared to the original QTL study (17.0 and 10.2 %), demonstrating that the more homozygous the background was, then the higher phenotypic variance could be explained. However, parthenocarpy is a complex trait that phenotypic data of a target individual can be influenced when fertilization is being conducted at the same time. Therefore, segregating population construction from one target individual can only be attained by cuttings, which make it difficult to produce enough seeds for further study before the coming planting season and fine mapping of this trait will take longer time. Currently we are developing a large segregating population by cuttings from the target individual to fine map this QTL.

### Linked markers as effective markers in MAS of parthenocarpy

Attaining closely linked marker is the prerequisite for MAS but not all of them can be well applied in breeding. Hence, maker validation before application is very important. Sun [33] found eight AFLP markers linked to parthenocarpy through QTL mapping whereas they were not validated and applied in cucumber breeding. In this study, we validated the effectiveness of the linked markers SSR16226, Indel-T-32, Indel-T-34 and Indel-T-39 with 99 F_3:4_ plants. It was also applied to 11 gynoecious and 10 monoecious cucumber inbred lines to test its accuracy. Among 11 gynoecious inbred lines, the extreme phenotype of parthenocarpic lines all showed the same genotype with corresponding parents, which demonstrated that the major-effect Parth2.1 does exist and play roles in extreme parthenocarpy materials. Whereas, all monoecious cucumber inbred lines showed low PP (Additional file 1: Table S4), and thus no relationship between the genotypes at these loci and the phenotype was observed. It probably due to fewer female flowers on monoecious plants produce less parthenocarpic fruits, or parthenocarpy in monoecious cucumber is controlled by different QTLs which need to be proved. As breeding parthenocarpic cultivars is labor intensive and time-consuming, these DNA markers will be effective tools for MAS in cucumber.

### Prediction of parthenocapic candidate genes

Mutations between the genes of EC1 and 8419 s-1 in CDS sequences have the potential for transcriptional or functional differences that can regulate parthenocarpic/non-parthenocarpic fruit set. In the present study, we found that 57 genes located in parth2.1 contains missense or frameshift mutations (Additional file 1: Table S6) including three phytohormone related genes. Auxin-dependent transcriptional regulation is mediated by regulatory proteins belonging to auxin/indole-3-acetic acid (AUX/IAA) and auxin response factor (ARF) families of transcription factors [51]. For example, ARF8, a member of *Arabidopsis* ARFs family, negatively regulates fruit set and leads to parthenocarpy in tomato and *Arabidopsis* by genetic alterations of *ARF8* function [52, 53]. In tomato, *Solanum lycopersicum* ARF7 (*Sl*ARF7) acts as a negative regulator of fruit set and transgenic plants with decreased *Sl*ARF7 mRNA levels forms seedless (parthenocarpic) fruits [18]. Since Csa2M068680 (*CsARF19*) is homologous to a member of *Arabidopsis ARFs*, *ARF19*, this indicates that it is a promising candidate gene involved in auxin signaling and it may trigger parthenocarpy. Another gene, Csa2M070230 (*CsWD40*), is an ortholog of *Arabidopsis WD40* that plays a role in cytokinin responses [38, 39]. It is also a promising candidate gene related to parthenocarpy because cytokinin is another phytohormone that can induce parthenocarpy [9, 54, 55]. Moreover, a reduction of ethylene production in the zucchini flower is able to induce fruit set and early fruit development, and therefore ethylene is actively involved in fruit set and early fruit development [56]. Csa2M070880 (*CsEIN1*) is an ortholog of *Arabidopsis ETHYLENE INSENSITIVE 1*(*EIN1*) that negatively regulates ethylene-activated signaling pathway [57–59]. This indicates that *CsEIN1* is also a promising candidate gene possibly involved in ethylene signaling pathway, and may result in parthenocarpy.

Previous studies in our lab suggested that endogenous hormones in the ovaries of EC1 maintain low levels during the process of fruit formation and development. There is a possibility that EC1 displays a hormone insensitive parthenocarpic fruit set [43]. So we did not exclude five non-phytohormone related genes, *CsPPR*, *CsHEXO3*, *CsMDL, CsDJC77* and *CsSMAX1* as candidate parthenocarpy genes because of their different expression patterns during parthenocarpic fruit set and fruit abortion (Fig. 6). Furthermore, more evidences are necessary to confirm the exact parthenocarpy genes and the mechanism of parthenocarpic fruit set of EC1 is remained to uncover in future study.

### Conclusion

We identified a major-effect QTL Parth2.1 and six minor-effect QTLs that contribute to the phenotypic variation of parthenocarpy in cucumber. Whole genome re-sequencing of two parents is an efficient method for development of polymorphic DNA markers and prediction of candidate genes. The marker closely linked to the Parth2.1 is an effective tool for MAS of parthenocarpy in cucumber. Results from this study improve our understanding of the possible genetic mechanisms that give rise to parthenocarpy in cucumber, and will provide guidance in manipulating it by biotechnology-assisted improvement.

## Methods

### Plant materials and an evaluation of expression of parthenocarpy

An F_2_ population including 145 plants, as well as F_2_-derived F_3,_ developed from a cross between two inbred lines EC1 and 8419 s-1 were used to map QTLs of parthenocarpy in cucumber. EC1, a gynoecious parthenocarpic inbred line was derived from a European greenhouse type ‘Delta star’. 8419 s-1, a monoecious non-parthenocarpic inbred line, originated from a European greenhouse type ‘Thamin beit alpha’.

**Fig. 7:**
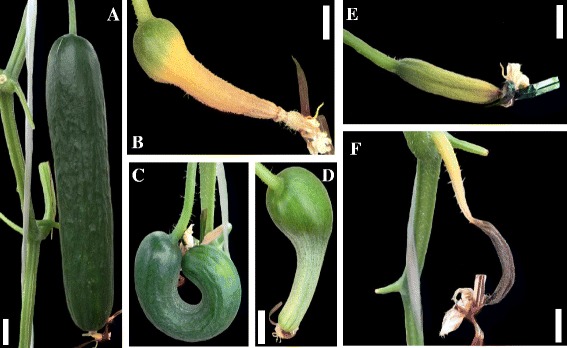
Situation of trapped cucumber in plastic house. **a** normal parthenocarpic fruit; **b**, **c** and **d** malformed parthenocarpic fruits; **e** and **f** aborted fruits. Scale bar indicates 10 mm

Phenotypic data were collected from 145 F_2:3_ families plus two parents and their F_1_ with ten plants each in spring and fall 2013 respectively in plastic houses at the Jiangpu Experiment Farm of Nanjing Agricultural University. Plants were only planted in four lines of two ridges in the middle of each plastic house and one ridge at each edge were left for other cucumber plants. Individual plants were spaced 30 cm apart and placed 80 cm apart in rows. Both seasons used the same complete randomized design (CRD). Each family planted 10 plants which were put next to each other. One day prior to anthesis, on each plant, we trapped eight female flowers from the fifth node above the main stem and eight more from the laterals with colorful metal wire. Well-developed (Fig. 7a) and malformed (Fig. 7b, c, d) fruits 10 days after trapping were counted to be parthenocarpic fruit, whereas aborted ones (Fig.7e, f) were non-parthenocarpic. Parthenocarpy percentage (PP): the ratio of parthenocarpic fruits to total trapped female flowers. An arcsin transformation of PP was used for QTL detection. We collected phenotype data on 130 families in the spring and 125 families in the fall without disease infection which were used for QTL analysis. The number of intersection family is 123 and data of these families were used for ANOVA. All phenotype data were arsin transformed.

Statistical analysis of phenotypic data was conducted with the software Statistical Analysis System (SAS V8). Analysis of variance (ANOVA) was performed with PROC VARCOMP function to estimate the genetic and season effects with a model like Y_ijk_ = mu + Family_i_ + Season_j_ + Family x Season_ij_ + error_ijk_. Y is observed value for parthenocarpy, mu grand mean. Broad sense heritability (*h*^2^_B_) estimate was calculated from variance components. The broad sense heritability was estimated using *h*^2^_B_ = σ^2^_F_/(σ^2^_F_ + σ^2^_FS_/Rs + σ^2^_E_/RsRn), where σ^2^_F_ was the family variance, σ^2^_FS_ was the family × season interaction (F × S) variance, and σ^2^_E_ was the residual variance, respectively. Rs was the number of seasons and Rn was the mode of individuals in each family. Correlations between PP in spring and fall were estimated using the PROC CORR function on the basis of PP means for each F_2:3_ family.

### Whole genome re-sequencing of both parents

DNA extraction of EC1 and 8419 s-1 was performed by the CTAB method. We constructed 500 bp paired-end sequencing libraries using genomic DNA ≥ 5ug from each parent, and sequenced these libraries using an Illumina Hiseq™ 2000. The raw data obtained by re-sequencing were processed to obtain clean data. The quality of these clean data was evaluated based on reads quantity, data output, error rate, and the content of Q20, Q30 and GC (Additional file 1: Table S5). The qualified data from two parents were aligned to reference the genome ‘9930’ separately after assessment, and then SAMTOOLS software [60] was used to delete duplications and identify single nucleotide polymorphisms (SNPs) and InDel (<50 bp) between EC1 and 8419 s-1.

### Genetic map construction

A set of 1335 cucumber SSR markers [61, 62] and 173 InDel markers were used for polymorphism screening between EC1 and 8419 s-1. InDel markers were designed with Primer Premier 5.0 software based on the re-sequencing data from both parents. Polymorphic markers were used to genotype 145 F_2_ plants. Descriptions of the polymorphic markers used for map construction are presented in Additional file 1: Table S2. Genomic DNA extraction followed the methods outlined above. The total volume of PCR is 10 μl containing 10 × buffers with Mg^2+^, 200 μM dNTP, 0.25 μM of each primer, and 0.5U Taq polymerase, 25 ng of template DNA. PCR amplification was performed at 94 °C /5 min for denaturation, followed by 35 cycles of denaturation at 94 °C/30s, annealing at 58–60 °C/30s, extention at 72 °C/80s, and the last extension step at 72 °C/10 min. The PCR products were separated on 7 % non-denaturing polyacrylamide gels and manually scored after silver staining. *χ*^2^ tests were run on each marker to examine deviation from the expected 1:2:1 segregation ratio. A genetic map was constructed using JoinMap 4.0 software with a minimum LOD score of 5.0 and the Kosambi mapping function.

### QTL detection and confirmation of the major-effect QTL Parth2.1

QTL detection for parthenocarpy in cucumber was performed using the arcsin transformed PP means of each F_2:3_ family in spring and fall 2013. QTL analysis was conducted with composite interval mapping (CIM) procedure within Windows QTL Cartographer v2.5 software [63]. The parameter setting was 1000 permutation tests at 1.0 cM walk speed and threshold at *P* ≤ 0.05. An LOD score of 2.5 was used to determine the presence of QTL. Nomenclature of a QTL was an abbreviation of the trait, followed by relevant chromosome number then QTL serial number on this chromosome.

One hundred and thirty five F_2:4_ families, each consisting of ten individuals, were planted in Pailou Experimental Greenhouse of Nanjing Agricultural University in winter 2013 in order to screen residual heterozygous plants. RHL97-5 segregated from a residual heterozygous plant 97–5 that is heterozygous for the major-effect QTL region between SSR marker SSR00684 and SSR22083 but homozygous for the other minor-effect QTLs. The RHL97-5 containing 161 plants was used to confirm the major-effect QTL. All markers in the target area (SSR00684-SSR22083) were used to genotype the 161 plants. Moreover, phenotype data collections of these plants were conducted as well. Linkage mapping analysis was performed based on high resolution linkage map and parthenocarpic phenotype data of the 161 plants. These plants were classified into three groups such as homozygous EC1, 8419 s-1 genotype and heterozygous ones based on the genotype in the target area (SSR00684-SSR22083), and ANOVAs were conducted among these three classes.

### Validation of the effectiveness of markers linked to Parth2.1

To evaluate the markers linked to Parth2.1, we planted 99 F_3:4_ plants derived from F_2:3_ family in the spring of 2014 and genotyped them with Indel-T-32, Indel-T-34 and two flanking markers, SSR16226 and Indel-T-39. Genotypes of these four markers (homozygous EC1, heterozygous and homozygous 8419 s-1) and plant numbers of each groups based on PP (0–20 %, 21–40 %, 41–60 %, 61–80 %, 81–100 %) were used to conduct the test for independence of 3 × 5 table (*χ*^2^ test) in order to explore the relationship between these markers and parthenocarpy. ANOVAs of PP among groups in terms of marker genotypes were also performed with significance at *P* < 0.05. Meanwhile, twenty-one different geographic origins and sexual type cucumber inbred lines (Additional file 1: Table S4) were also used to genotype with these marker.

### Identification of candidate genes for the Parth2.1

Genes located within the confidence interval of Parth2.1 were analyzed based on the whole genome parental re-sequencing and transcriptome data. Coding sequences in Parth2.1 region were searched to detect mutated sequences between EC1 and 8419 s-1 using the SAMTOOLs. Only those genes causing amino acid changes were considered as candidate genes. *Arabidopsis* orthologous gene information for candidate genes was obtained from The *Arabidopsis* Information Resource (TAIR, http://www.arabidopsis.org/). Ovary samples of trapping-treated EC1 and 8419 s-1 at two days post anthesis (dpa) were harvested for RNA-seq analysis. The details about how the transcriptomics experiment was carried out have been presented by Li [43]. There were 3090 up-regulated and 2211 down-regulated differentially expressed genes (DEG) (the false discovery rate ≤ 0.001 and the fold ≥ 1.5) between these two samples. DEG within Parth2.1 between two parents were selected and their annotations are presented in Additional file 1: Table S7.

### RNA extraction and quantitative real-time PCR (qRT-PCR) analysis of DEG

Ovary samples of trapping-treated EC1 and 8419 s-1 at 0dpa, 2dpa, 4dpa were collected respectively for qRT-PCR. For each sample, 20 individual ovaries were ground into powder and mixed in liquid nitrogen (three replicates). Total RNAs were isolated using Trizol (Invitrogen) according to the manufacturer’s protocol and Rnase-free DNase I was used to remove DNA in RNA samples. cDNA was prepared with 2 μg of total RNA, using a cDNA Synthesis Kit (Fermentas). Quantitative real-time PCR was conducted with the SYBR Premix Ex Taq™ Kit (TAKARA) following the manufacturer’s instructions on a Bio-Rad CFX96 Real-Time PCR machine. The PCR program is: denaturation at 95 °C for 30 s and 40 cycles of 95 °C for 5 s and 60 °C for 30 s. Primers were designed using Primer Premier 5.0 software and Actin (GenBank ID: AB010922) was used as the internal control gene. The relative expression levels of each gene for different treatments were normalized to Actin gene and calculated with the 2-△△Ct method. The primers used for qRT-PCR are listed in Additional file 1: Table S7. Reactions for each gene and sample were performed with three repeats.

## Additional files

Additional file 1: Table S1. Analysis of variances (ANOVA) for parthenocarpy in F_2:3_ from two-season experiments **Table S2.** Information of SSR and InDel markers mapped on the EC1 × 8419 s-1 F_2_ genetic map and high-resolution map. **Table S3.** Statistics of genetic map constructed in this study. **Table S4.** Information of 21 inbred lines of cucumber. **Table S5.** Information of total reads, re-sequencing data quality and sequence variations of EC1 and 8419 s-1. **Table S6.** List of 57 candidate genes within the main-effect QTL Parth2.1 region. **Table S7.** Annotations and fold values of DEG within Parth2.1 and primers used for qRT-PCR. (XLS 269 kb) Additional file 2: Correlation analysis between PP for the F_2:3_ families in spring and fall in 2013. Parthenocarpy percentages were acsin transformed. (DOCX 63 kb) Additional file 3: Mapping of QTLs for parthenocarpy using an F_2_ population derived from a cross between EC1 and 8419 s-1. The position of five QTLs detected in spring are illustrated by *hollow black bars* next to the chromosome, while the position of three QTLs detected in fall are depicted by *solid black bars*. (DOC 188 kb)

## Abbreviations

AFLP: Amplified fragment length polymorphism
ANOVA: Analysis of variance
ARF: Auxin response factor
CIM: Composite interval mapping
DEG: Differentially expressed genes
InDel: Insertion/deletion
LOD: Logarithm of odds
MAS: Marker-assisted selection
PCR: Polymerase chain Reaction
PP: Parthenocarpy percentage
PVE: Phenotypic variance explained
qRT-PCR: Quantitative real-time PCR
QTL: Quantitative trait locus
RHL: Residual heterozygous line
SAS: Statistical analysis system
SNP: Single nucleotide polymorphism
SSR: Simple sequence repeats
TAIR: The *arabidopsis* information resource
