# Supplementary material for: Identification of a stable major-effect QTL (Parth 2.1) controlling parthenocarpy in cucumber and associated candidate gene analysis via whole genome re-sequencing
Source: BMC Plant Biol. 2016 Aug 23;16(1):182. doi: 10.1186/s12870-016-0873-6 (PMC4995632; doi:10.1186/s12870-016-0873-6)
Supplement: Additional file 2: — Correlation analysis between PP for the F2:3 families in spring and fall in 2013. Parthenocarpy percentages were acsin transformed. (DOCX 63 kb) [file 12870_2016_873_MOESM2_ESM.docx]

**Parthenocarpy percentages in spring**

**Parthenocarpy percentages in fall**
