# Supplementary figures and images for: Identification of a stable major-effect QTL (Parth 2.1) controlling parthenocarpy in cucumber and associated candidate gene analysis via whole genome re-sequencing
Source: BMC Plant Biol. 2016 Aug 23;16(1):182. doi: 10.1186/s12870-016-0873-6 (PMC4995632; doi:10.1186/s12870-016-0873-6)

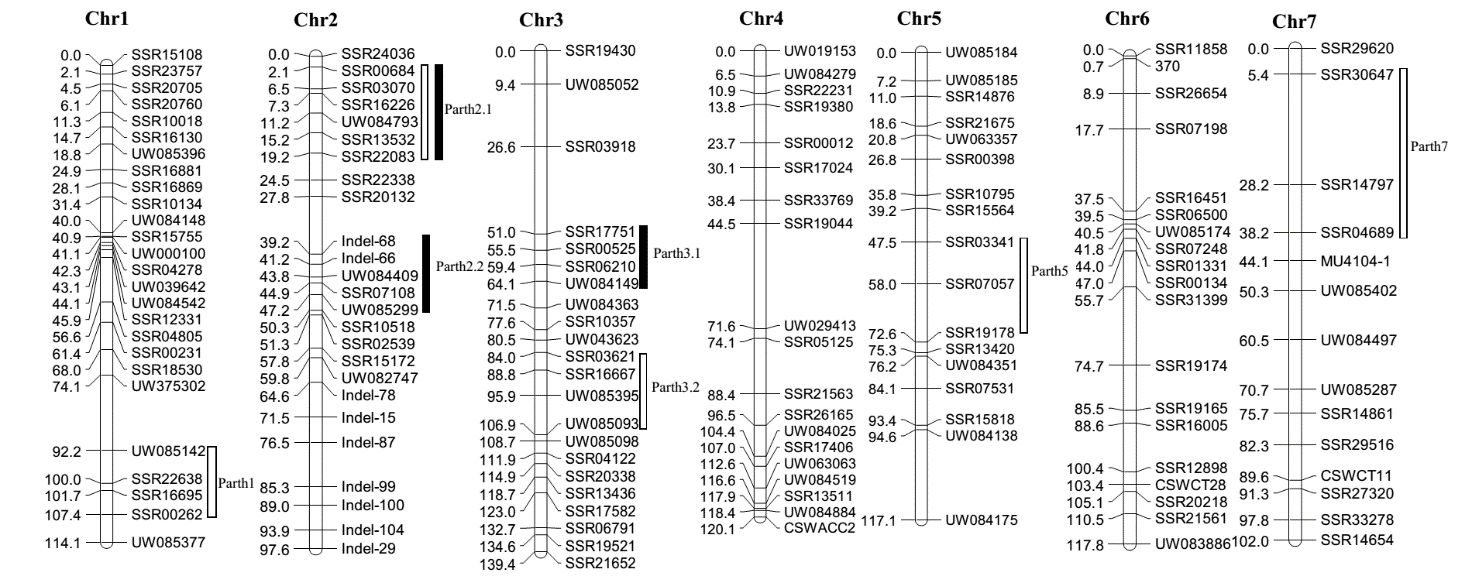

Supplement: Additional file 3: — Mapping of QTLs for parthenocarpy using an F2 population derived from a cross between EC1 and 8419 s-1. The position of five QTLs detected in spring are illustrated by hollow black bars next to the chromosome, while the position of three QTLs detected in fall are depicted by solid black bars. (DOC 188 kb) [file 12870_2016_873_MOESM3_ESM.doc]
